# Obesity and Bone Loss at Menopause: The Role of Sclerostin

**DOI:** 10.3390/diagnostics11101914

**Published:** 2021-10-16

**Authors:** Paolo Marzullo, Chiara Mele, Stefania Mai, Antonio Nardone, Massimo Scacchi, Gianluca Aimaretti

**Affiliations:** 1 Department of Translational Medicine, University of Piemonte Orientale, 28100 Novara, Italy; chiara.mele1989@gmail.com (C.M.); gianluca.aimaretti@med.uniupo.it (G.A.); 2 Istituto Auxologico Italiano, IRCCS, Division of General Medicine, S. Giuseppe Hospital, 28824 Piancavallo, Italy; massimo.scacchi@unimi.it; 3 Laboratory of Metabolic Research, Istituto Auxologico Italiano, IRCCS, S. Giuseppe Hospital, 28824 Piancavallo, Italy; s.mai@auxologico.it; 4 Department of Clinical-Surgical, Diagnostic and Pediatric Sciences, University of Pavia, 27100 Pavia, Italy; antonio.nardone@icsmaugeri.it; 5 Neurorehabilitation and Spinal Unit, Istituti Clinici Scientifici Maugeri SPA SB, Institute of Pavia, IRCCS, 27100 Pavia, Italy; 6 Department of Clinical Sciences and Community Health, University of Milan, 20122 Milan, Italy

**Keywords:** obesity, menopause, osteoporosis

## Abstract

**Background**. Peripheral fat tissue is known to positively influence bone health. However, evidence exists that the risk of non-vertebral fractures can be increased in postmenopausal women with obesity as compared to healthy controls. The role of sclerostin, the SOST gene protein product, and body composition in this condition is unknown. **Methods**. We studied 28 severely obese premenopausal (age, 44.7 ± 3.9 years; BMI, 46.0 ± 4.2 kg/m^2^) and 28 BMI-matched post-menopausal women (age, 55.5 ± 3.8 years; BMI, 46.1 ± 4.8 kg/m^2^) thorough analysis of bone density (BMD) and body composition by dual X-ray absorptiometry (DXA), bone turnover markers, sclerostin serum concentration, glucose metabolism, and a panel of hormones relating to bone health. **Results**. Postmenopausal women harbored increased levels of the bone turnover markers CTX and NTX, while sclerostin levels were non-significantly higher as compared to premenopausal women. There were no differences in somatotroph, thyroid and adrenal hormone across menopause. Values of lumbar spine BMD were comparable between groups. By contrast, menopause was associated with lower BMD values at the hip (*p* < 0.001), femoral neck (*p* < 0.0001), and total skeleton (*p* < 0.005). In multivariate regression analysis, sclerostin was the strongest predictor of lumbar spine BMD (*p* < 0.01), while menopausal status significantly predicted BMD at total hip (*p* < 0.01), femoral neck (*p* < 0.001) and total body (*p* < 0.05). Finally, lean body mass emerged as the strongest predictor of total body BMD (*p* < 0.01). **Conclusions**. Our findings suggest a protective effect of obesity on lumbar spine and total body BMD at menopause possibly through mechanisms relating to lean body mass. Given the mild difference in sclerostin levels between pre- and postmenopausal women, its potential actions in obesity require further investigation.

## 1. Introduction

Despite being acknowledged as beneficial to bone mineral density (BMD) due to the mechanical loading effect of weight excess [1], obesity is emerging as a potential detrimental factor for bone health, particularly appendicular bones [2]. Studies in adolescents and adults pinpointed the negative effects of body fat excess on bone strength [3,4,5] and cortical rearrangement through insulin resistance [6]. In single-center analysis, osteoporosis was associated with obesity in one out of three women [7], while prospective studies found that nearly one out of four postmenopausal women with fractures presented with obesity, with obesity acting as the dominant risk factor for ankle and upper leg fractures [8]. In parallel, obese postmenopausal women were found to harbor reduced femoral neck BMD and increased risk of non-vertebral fragility fractures [9].

Reid [10] proposed the common stromal cell origin of osteoblasts and adipocytes as a possible link between adipose tissue and bone. An interaction between obesity, menopause and bone metabolism exists [11] and involves several potential mechanisms: menopauses promotes visceral fat accumulation [12] and sarcopenia [13], which compromise the mechanical loading effect [14]; fat accumulation lowers vitamin D levels with secondary hyperparathyroidism, which results in bone loss and accelerated osteoporosis [15]; obesity impacts endocrine signals active on the bone, such as the somatotroph [16], adrenal [17] and thyroid axis [18]; type 2 diabetes mellitus (T2DM) impairs the strength of femoral neck relative to mechanical load [19] and enhances the fracture risk [20]. It is noteworthy that leptin and other adipokines secreted from the adipose tissue can modulate bone cells through major inhibition of bone remodeling, whereas molecules activating the peroxisome proliferator-activated receptor-γ can drive mesenchymal stem cell differentiation from osteoblastic towards adipocyte lineage [21]. It is known that obesity is characterized by leptin resistance, which has been linked to decreased bone mass, as it happens in case of extreme leanness with hypoleptinemia [22]. Moreover, insulin partakes in the feedback loop between pancreas and osteoblasts [23], enhances bone resorption and promotes the decarboxylation of osteocalcin, a bone-derived protein that is capable of regulating insulin release, insulin sensitivity, and fat mass accrual [24]. Finally, the insulin-sensitizing adipocyte-derived protein adiponectin can modulate bone remodeling via osteocalcin as well [25].

Among the intermediate pathways, the Wnt/β catenin signaling pathway is known to stimulate the expansion of osteoprogenitor cells and suppress apoptosis of mature osteoblasts and osteoclastogenesis in response to biomechanical stress [26]. Wnt also plays a role in adipocyte differentiation and pathogenesis of metabolic diseases [27]. Importantly, Wnt is antagonized by the osteocyte-secreted product of the SOST gene, sclerostin [28,29]. Sclerostin predicts bone loss in relation to age, gender and menopause [30], prolonged immobilization [31], and postmenopausal hip fracture risk [32]. Studies also emphasized the potential role of sclerostin in relation to adiposity and type 2 diabetes mellitus [33,34]. While no difference in serum sclerostin was documented in obese as compared to control women [35], serum sclerostin was found to be negatively associated with insulin sensitivity in obese but not lean subjects, suggesting a potential role for the Wnt/β-catenin pathway in regulating insulin sensitivity in obesity [36]. Moreover, sclerostin has been found to increase in states of unloading and to possibly mediate the changes in bone metabolism associated with weight loss and exercise [37], and population studies reported a negative association between sclerostin and skeletal muscle mass after adjusting for confounding factors, including age, sex, bone mineral content, and total body fat mass [38].

Based on the peculiar postmenopausal osteoporosis risk in obesity and the involvement of the Wnt pathway in the link between osteogenesis and adipogenesis, we sought to explore the role of circulating sclerostin on skeletal bone in pre- and post-menopausal women in relation to body composition, glucose homeostasis and a comprehensive set of bone/adipose tissue markers in women with obesity. Secondly, we aimed to assess the control operated by these factors on circulating sclerostin to profile its determinants in the obese state.

## 2. Materials and Methods

### 2.1. Study Population

This study included 56 women with severe obesity (BMI > 35 kg/m^2^) enrolled as premenopausal (n = 28) and postmenopausal subjects (n = 28) (Table 1). Menopause was defined as cessation of menstrual bleeding for at least 12 months together with low estradiol and high FSH levels. In our postmenopausal subgroup, mean menopause duration was 5.1 ± 5.1 years. All participants enrolled in the study had long-lasting obesity (>10 years), and obesity was defined as having a BMI > 30 kg/m^2^. The exclusion criteria for the study included: menarche > 16 years; previous hysterectomy, ovarian surgery, sex hormone replacement treatment, menopause < 45 years; osteoporosis treatment; previous diagnosis of type 1 diabetes mellitus (T1DM) and T2DM, autoimmune disorders affecting bone metabolism; chronic steroid, heparin or anti-convulsant therapy; evidence of spontaneous fractures, osteogenesis imperfecta, family history of severe osteoporosis malnutrition, malabsorption, chronic liver or kidney disease; less than one alcoholic drink reported per day and smoking habit. Each participant was admitted to the study as part of a regular workup of obesity and its complications, and enrolled after signing an informed consent. The study was conducted in accordance with the Declaration of Helsinki, and the protocol was approved by the Ethics Committee of Istituto Auxologico Italiano.

### 2.2. Methods

Each subject underwent anthropometric measurements, routine blood and urine analysis, baseline hormone study followed by the oral glucose tolerance test (OGTT), overnight dexamethasone oral suppression test (OST) and dual X-ray absorptiometry (DXA) on separate days and in fasting conditions.

Blood glucose, electrolytes and glycated haemoglobin (HbA1c) were measured by enzymatic methods (Roche Molecular Biochemicals, Mannheim, Germany). A two-site, solid-phase chemiluminescent immunometric assay or competitive immunoassay was used for insulin, FSH, LH, FT4, TSH, GH, PRL, IGF-I, estradiol, testosterone, cortisol, DHEAS, osteocalcin and PTH levels (Immulite 2000 Analyzer; DPC, Los Angeles, CA, USA). Chemiluminescence was used for measurement of bone alkaline phosphatase (BAP) with intra and interassay coefficients of variation (CVs) of 4% and 6.1%, respectively (Liaison Bap Ostase, Diasorin, Stillwater, MN, USA); and carboxy-terminal telopeptide of type I collagen (CTX), with intra and interassay CVs of 3.5% and 2.2% (Elecsys, Roche Diagnostics GmbH, Mannheim, Germany). Enzyme immunoassays were used for measurement of urine amino-terminal collagen cross-links (NTX), with intra and interassay CVs of 4.6% and 6.9% (Wampole Laboratories, Princetown, NJ, USA); sclerostin levels, with intra and interassay CVs of 5% and 3% (Biomedica; Wien, Austria); osteocalcin levels, with intra and interassay CVs of 3% and 4%; adiponectin levels, with intra and interassay CVs of 7% and 8.4% (DRG Instruments GmbH, Marburg, Germany); leptin levels, with intra- and interassay CVs of 5.9% and 6.9% (Linco, St. Louis, MO, USA). Levels of 25OH-Vitamin D were measured using a Cobas Integra 800 Autoanalyzer (Roche, Indianapolis, IN, USA). Upon OGTT, ADA guidelines were applied for glucose tolerance [39] as follows: normal fasting plasma glucose (FPG) if <100 mg/dL (5.6 mmol/L); impaired FPG if 100–125 mg/dL (6.9 mmol/L); impaired glucose tolerance (IGT) if 2h OGTT-PG 140–199 mg/dL (7.8–11.0 mmol/L); T2DM if FPG > 126 mg/dL (7 mmol/L) on two days apart or if 2-h OGTT-PG > 200 mg/dL (>11.1 mmol/L). HbA1C values of 5.7 and 6.4% were considered as threshold for normal glucose and T2DM, respectively. Insulin resistance was calculated by the homeostatic model of insulin resistance (HOMA-IR) as fasting insulin (μU/m) × [fasting PG (mmol/L)/22.5].

### 2.3. Bone Mineral Densitometry and Body Composition Analysis

BMD measurements of the lumbar spine (L1–L4) and proximal femur were undertaken with the Prodigy densitometer (Lunar, Madison, WI, USA). Body composition was measured as derivative values of lean body weight and total body percentage fat. A positioning device was used to facilitate the reproducible measurement of the proximal femur. Quality control by daily measurement of an anthropomorphic spine phantom at each site, calibration with a spine phantom to provide cross-site and cross-time calibrations, and a site-level review of all participant scans for specified criteria. Bone scans were acceptable if there was no radiological interference from truncal adiposity, arm or leg did not overlap, no body parts were outside the scan field or positioning problems due to adiposity, no motion. In the case of the proximal femur, all femur subregions had to be valid in order for data to be retained for any of the subregions. Because the three BMD measures correlated highly with each other, a value of total BMD was incorporated in the analysis by averaging all sites analyzed.

### 2.4. Statistical Analysis

All data are expressed as mean ± SD. Data were tested for normality of distribution by the Shapiro–Wilk test and log-transformed when needed, to correct for skewness. Differences between pre- and postmenopausal subjects were calculated by two-tailed unpaired *t* test. Correlations analyses were calculated with the Pearson’s coefficient. Inspection of the distributions of the variables indicated that the two groups overlapped. Therefore, to make the correlation analysis numerically more consistent, Pearson correlations were performed on the total 56 subjects to assess relationships among variables. The general linear model and analysis of covariance (ANCOVA) were used to evaluate the interaction between variables after statistically controlling for the effects of menopause; effect sizes and interactions were computed between variables and the covariate. Non-collinear independent predictors were included in a stepwise multiple regression model, as described in the results section. For significance, *p* < 0.05 was considered of statistical value. Analyses were performed with the SPSS 21.0 (SPSS, Inc., Chicago, IL, USA).

## 3. Results

Table 1 illustrates the large set of bone/adipose tissue markers analyzed in this study. With the exception of the expected differences in menopause-related hormones, menopause did not significantly alter the hormone profiles investigated herein, while adiponectin levels were higher after menopause possibly due to an age-related effect. In terms of glucose metabolism, premenopausal and postmenopausal women with obesity exhibited comparable rates of IFG (53.5% vs. 35.7%), IGT (35.7% vs. 32.1%) and T2DM (25% for both).

Analysis of bone-related parameters only revealed between-group differences in CTX and NTX levels while sclerostin levels showed a trend toward an increase after menopause (Table 2). The rate of vitamin D deficiency (<20 mcg/L) was similar between pre- and postmenopausal women (92.8% vs. 96.4%). In women with newly diagnosed T2DM, we documented lower levels of osteocalcin (3.8 ± 2.1 vs. 6.7 ± 3.7 μg/L, *p* < 0.01), CTX (234 ± 89.1 vs. 376.9 ± 140.3 pg/mL, *p* < 0.01) and NTX levels (27.2 ± 7.8 vs. 37.6 ± 15.9 nM/BCE, *p* < 0.05) as compared to their non-diabetic counterpart.

DXA scanning revealed lower lean mass and higher fat mass in postmenopausal as compared to premenopausal women. Lumbar spine BMD was similar between groups, while lower BMD values at the total hip (−0.138 g/cm^2^), femoral neck (−0.147 g/cm^2^) and total skeleton (0.210 g/cm^2^) were documented in postmenopausal women compared to premenopausal ones (Table 3). BMD values suggestive of osteoporosis were only observed at the lumbar spine in one premenopausal woman.

**Figure 1:**
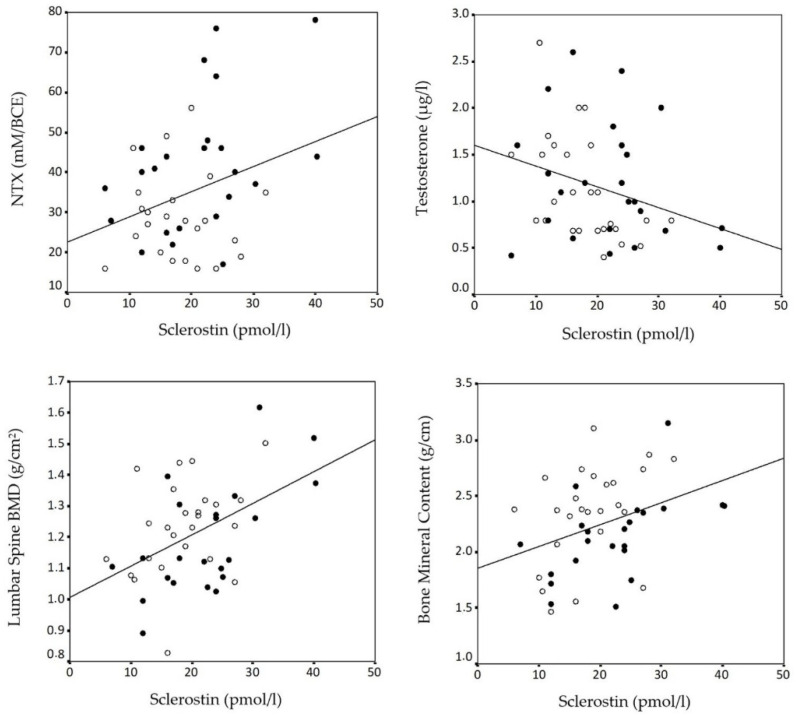
Bivariate correlation analysis between sclerostin levels (pmol/L) and NTX levels (mM/BCE), testosterone levels (µg/L), Lumbar Spine BMD (g/cm^2^) and BMC (g/cm) in premenopausal (open circles) and postmenopausal obese women (closed circles).

In correlation analysis (Table 4, Figure 1), sclerostin was associated with NTX and testosterone levels, as well as with lumbar spine BMD and skeletal BMC, while an expected inverse association related estradiol with NTX (r = −373, *p* < 0.01) and CTX levels (r = −290, *p* < 0.05). Lean mass clearly elicited a protective effect on BMD at the femoral neck (r = 0.301, *p* < 0.05), total hip (r = 0.290, *p* < 0.05) as well as total body (r = 0.499, *p* < 0.001). ANCOVA showed that a poor interaction between lean mass and menopause on total BMD (F = 0.6, not significant).

As shown in Table 5, a multivariable model was built to test the role of hormone and adiposity markers on sclerostin levels and bone health. Sclerostin emerged as the strongest predictor of BMD at the lumbar spine. In turn, testosterone levels and menopause explained about 40% in the variability of sclerostin levels. As expected, menopause significantly predicted BMD at the femoral neck, total hip and total body. Similar correlations were obtained when estradiol levels replaced as a continuous variable the dichotomic menopausal status. Finally, lean body mass emerged as the strongest predictor of total BMD.

**Figure 2:**
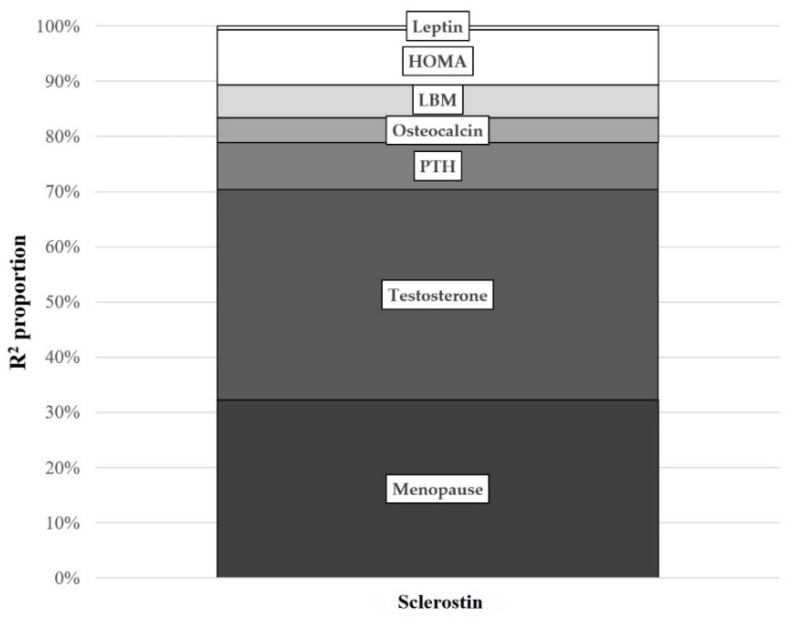
Total variance proportion (R^2^ proportion) observed for each variable with the use of an univariate analysis of variance in sclerostin prediction model according to η^2^ values.

We then assessed the determinants of circulating sclerostin in our obese cohort. Figure 2 presents η^2^ values that explain how much (the percentage) each variable contributed to the total variability in sclerostin levels. Testosterone levels and menopause explained the highest impact accounting together for 70% of circulating sclerostin variability, while a modest influence was observed for HOMA-IR and PTH concentrations.

## 4. Discussion

This comprehensive study conducted in severely obese women distinguished by menopause provides evidence that obesity does not associate with (early) postmenopausal bone loss at the lumbar spine, with sclerostin acting as the strongest protective determinant at this site. By contrast, menopause associates with unfavorable changes in femoral BMD, with the protective effect of lean mass being predominant at this site. Site-specific relations between obesity and bone health hence provide evidence of peculiar biochemical and anatomical determinants for such effects.

Menopause causes bone loss in some women more rapidly than in others. High body weight and BMI putatively act on bone composition and fracture risk protectively, with rates of spine and hip bone loss reported to be 35–55% lower in postmenopausal women having their body weight in the top tertile than those at the lowest tertile [40]. Potential explanatory mechanisms reside in extraovarian contribution of estradiol from aromatase activity in fat tissue [41] and mechanical loading effect caused by weight excess, which stimulates proliferation and differentiation of osteoblasts and osteocytes through the Wnt/β-catenin pathway [42,43]. However, considering that the degree of physical activity is less consistent in obese women, as it generally is in the postmenopausal state, it should be considered that obese women may be more exposed to peripheral fractures, given the prominent weight-bearing activity of the spine. Increasing credit is given to the suggestion that adiposity actually impairs bone health due to direct and indirect mechanisms relating to decreased ostoblastogenesis and increased adipogenesis [44]; increased osteoclast activity through proinflammatory cytokines and RANKL/RANK/OPG pathway [45], which regulates osteoclast formation, activation and survival in normal bone modeling and in a variety of pathologic conditions characterized by increased bone turnover [46]; degenerative and inflammatory disorders of the musculo-skeletal system [47]; excess in fat intake interfering with intestinal calcium absorption [48]; altered vitamin D and PTH status [15]. Furthermore, fat deposition in vertebral bone marrow influences bone mass and fracture risk [49]. In pre-and postmenopausal cohorts, fat mass has been found inversely associated with BMD and correlated with non-spine fractures, when body weight is kept constant [50]. Also, obesity increased the incidence of ankle and upper leg fractures in postmenopausal women [8]. Finally, sarcopenic obesity with a predominant visceral phenotype has been found associated with a greater fracture risk, in addition to promote proinflammatory and dysmetabolic changes [51]. In this comprehensive study, we examined: (1) the overall effect of early menopause on bone density in relation to metabolic and hormonal profiles linked to obesity and bone health; (2) the involvement of sclerostin in specific-site mineral content; and (3) the role of body composition on bone health across menopause.

*Bone density and metabolism:* In perimenopausal women, BMD of the radius, femoral neck, and spine declines progressively [40]. Parallel results have been underscored in women spanning a wide age range, where postmenopausal bone loss was found to be significant, both at the lumbar spine and hip [52] but more so at the Ward’s triangle [53]. The Study of Women’s Health Across the Nation (SWAN) outlined an accelerated bone loss during 1 year preceding and 2 years following the final menses at the lumbar spine, where BMD decreased by 3.2–5.6% in the late perimenopause and after menopause [54], respectively, while milder bone loss was observed at the hip [40,54]. In the present series, postmenopausal obesity did not change lumbar spine BMD, while it associated with a 12% and 13% lower BMD at the hip and femoral neck, respectively, as compared to the premenopausal state. Given the coordinated changes seen in bone-specific markers, anabolic hormones and lean mass, this site-specific effect of (early) menopause in obesity suggest an impairment in cortical porosity and cortical thickness as the potential consequence of menopause in obesity. These changes may alter the mechanosensory properties of bone tissue and local tissue repair responses, which progress to microdamage with aging [55], a circumstance that is consistent with the higher rates of ankle and upper leg (fragility) fractures seen in postmenopausal obesity [8,9]. Biochemical dynamics consistent with increased bone turnover in menopause involved increments in circulating CTX and NTX. Such increase, which has been already seen in obesity [35,56], predicts the rate of bone loss in postmenopausal women and the risk of osteoporosis in elderly women [57]. Collagen telopeptides were also related to osteocalcin, a non-collagen protein of bone matrix and one of the osteoblast-specific proteins, which was in turn oppositely associated to total body BMD. This relationship extends to obesity the role of osteocalcin as a marker of bone turnover rather than a specific marker of bone formation, as it functions to limit bone formation without impairing bone resorption or mineralization [58]. In our hands, an inverse association related osteocalcin and glucose metabolism, which could agree with the antagonizing effect of osteocalcin on hyperglycemia and β-cell dysfunction [59] and substantiate the converging effect of dysmetabolic changes of obesity with bone health. As such, a negative association related insulin resistance to BMC, which agrees with evidence of increasing insulin resistance across with decreasing BMC [4]. Insulin resistance may thus reflect a cardinal pathway promoting lower bone strength and increased risk of fracture in patients with diabetes mellitus [6,19].

*Sclerostin and bone-regulating hormones:* Being a factor that promotes bone resorption, we evaluated sclerostin and its regulation operated by perimenopausal hormone status so as to identify potential modulators of bone status in obesity. Previous studies found that sclerostin levels increase with age [60], menopause [61], insulin resistance and type 2 diabetes mellitus [34], while a debated association with obesity exists [35]. In our population, mean sclerostin levels were slightly increased in postmenopausal women, a finding that agrees with studies showing that sclerostin progressively increases after menopause [62]. With regard to sex steroids, it is known that estrogens suppress circulating and bone sclerostin mRNA levels while an opposite role has been observed for testosterone both in experimental and clinical studies [30,63,64]. In our hands, there was no association between estradiol and sclerostin levels while, surprisingly, sclerostin decreased with increasing testosterone. As such, menopause (positively) and testosterone (negatively) largely predicted sclerostin variability in multivariable regression analysis. We speculate that the negative control exerted by testosterone on sclerostin levels is peculiar of obesity and likely due to enhanced fat-derived androgen aromatization. It is known that obesity associates with variable androgen excess throughout the lifespan both in pre- and post-menopausal women, which does not often manifest with specific clinical signs or symptoms and originates from changes in the pattern of secretion and/or metabolism, transport, and/or local action of androgens [65,66,67]. With regard to bone health, sclerostin levels were found to be positively associated with lumbar spine BMD, a finding that was confirmed in multivariable regression analysis. This correlation expands to obesity similar findings previously described in different cohorts [30,32,33,68,69], although it seems to conflict with the positive association between sclerostin and NTX levels seen herein, as well as with the intrinsic osteopenic effects of sclerostin [26,28]. Further studies are required to clarify this issue.

Within the comprehensive hormone panel screened in clinical setting, we failed to identify a role for vitamin D, adiponectin and leptin, as well as the somatotroph and thyroid axes, on bone health. Thus, it is conceivable that other peripheral factors, such as body composition, may play a role in bone health in obesity.

*Body composition:* While lean body mass was decreased in postmenopausal women, percent fat body mass was similar between groups. Total body BMD was directly associated to lean body mass and BMI, whereas the association to body fat was the opposite. Furthermore, lean mass and menopause acted as independent predictors of total body BMD and explained nearly 50% of its variability. These findings highlight the prevalent role of dynamic biomechanical forces over passive loads on bone composition in obesity. Previous studies in adolescents and young women have linked bone strength to the dynamic loads from muscle force rather than fat mass [70,71]. In Chinese cohorts, the percentage of body fat was related to the risk of osteoporosis, osteopenia, and non-spine fractures independent of body weight [50]. Studies in different ethnicities confirmed that percent fat mass was inversely related to weight-adjusted bone mass after removal of age, sex, menopause status, exercise, and smoking [72]. Intriguingly, visceral fat accumulation was found to negatively predict femoral cross-sectional area, cortical bone area, principal moment maximum, principal moment minimum, and polar moment, while subcutaneous acted as a positive correlate [5]. Together, these findings corroborate the link between body fat and limb fractures in obese postmenopausal women.

*Caveats of the study:* This study has some limitations. Since our investigation included severely obese Caucasian women in the perimenopausal age range, these findings cannot be generalized to other contexts or be extended to all obese women. The study design did not include a control group, due to the obese-restricted analysis of bone metabolism across menopause. Because of the cross-sectional design, any alteration would be random with respect to case status, and could probably underestimate the observed associations. We believe that the strict inclusion criteria and population selection of the study constitute a point of strength. Indeed, a future menopause transition study will appropriately reduce the risk of biased detection. Also, calcium intake and nutritional habits were not taken in appropriate account. Lastly and more notably, DXA has technical limitations in obesity associated with extraosseous soft tissue composition, so that BMD will appear to decrease more slowly in subjects with more soft tissue fat and vice versa [73]. Increased soft-tissue inhomogeneity is likely to occur from a greater and/or more variable amount of visceral fat surrounding the organs and subcutaneous fat around the hips in overweight and obese women. Increased percent body fat increases BMD precision errors, particularly at the lumbar spine and femoral neck regions of interest [74].

## 5. Conclusions

This cross-sectional analysis suggests that obesity protects the lumbar spine from bone loss caused by menopause possibly through pathways involving sclerostin. The positive association seen between total body bone density and lean body mass offers a potential clue for preventive measures against osteoporosis in this setting. Menopause-transition studies are warranted to better discriminate the reasons for such selective changes in obesity.

## Figures and Tables

**Table 1:** Anthropometric and biochemical parameters measured in the study groups. Significance was calculated by two-tailed unpaired Student’s *t*-test. For significance: * *p* < 0.05; ** *p* < 0.01; *** *p* < 0.001.

Parameters	Premenopausal Women (N = 28)	Postmenopausal Women (N = 28)
Age (years)	44.7 ± 3.9	55.5 ± 3.8 ***
Weight (kg)	117.2 ± 9.7	113.7 ± 13.6
Height (cm)	158.8 ± 7.2	157.0 ± 6.3
BMI (kg/m^2^)	46.0 ± 4.2	46.1 ± 4.8
Glucose (mg/dL)	102.0 ± 16.0	111.9 ± 32.0
Post-OGTT glucose (mg/dL)	154.7 ± 62.2	146.6 ± 54.6
Insulin (mIU/mL)	14.8 ± 8.4	14.1 ± 8.5
Post-OGTT insulin (mIU/mL)	103.6 ± 82.7	88.1 ± 55.4
HbA1c (%)	6.1 ± 0.5	6.5 ± 1.4
HOMA-IR	3.8 ± 2.4	3.8 ± 2.3
Leptin (μg/L)	75.5 ± 21.8	78.8 ± 29.7
Adiponectin (μg/L)	8.1 ± 3.0	11.2 ± 5.3 **
FSH (μg/L)	6.5 ± 5.4	45.7 ± 22.3 ***
LH (μg/L)	5 ± 4.3	25.9 ± 10.7 ***
PRL (μg/L)	21.5 ± 11.3	13.4 ± 8.8 *
Estradiol (μg/L)	110.1 ± 80	40.2 ± 12.3 ***
Testosterone (μg/L)	1.1 ± 0.5	1.2 ± 0.6
Free T4 (pg/mL)	10.6 ± 2.1	10.6 ± 2.6
TSH (mU/l)	2.2 ± 0.9	2.3 ± 2.1
GH (μg/L)	0.3 ± 0.6	0.3 ± 0.3
IGF-I (μg/L)	116.4 ± 46.9	110.0 ± 43.6
DHEA-S (μg/L)	3.2 ± 1.6	1.7 ± 1.0 ***
OST (μg/L)	1.2 ± 0.9	1.6 ± 2.2

Abbreviations: BMI, body mass index; OGTT, oral glucose tolerance test; HbA1c, hemoglobin A1c; HOMA-IR, homeostasis model assessment-estimated insulin resistance; FSH, follicle-stimulating hormone; LH, luteinizing hormone; PRL, prolactin; TSH, thyroid-stimulating hormone; GH, growth hormone; IGF-I, insulin-like growth factor-1; DHEA-S, dehydroepiandrosterone sulfate; OST, overnight dexamethasone oral suppression test.

**Table 2:** Bone-related biochemical and hormone parameters measured in the study groups. Significance was calculated by two-tailed unpaired Student *t* test. For significance: ** *p* < 0.01; *** *p* < 0.001.

Parameters	Premenopausal Women (N = 28)	Postmenopausal Women (N = 28)
Calcium (mg/dL)	9.0 ± 0.4	9.1 ± 0.5
Phosphate (mg/dL)	3.7 ± 0.5	3.8 ± 0.6
CTX (pg/mL)	293.6 ± 103.7	399.1 ± 160.9 ***
NTX (nM/BCE)	28.7 ± 10.5	41.3 ± 16.4 ***
BAP (μg/L)	13.2 ± 6.0	20.9 ± 22.4 **
PTH (μg/L)	74.6 ± 28.2	74.4 ± 34.9
25OH-vitamin D (μg/L)	11.0 ± 7.4	9.6 ± 7.2
Sclerostin (pmol/l)	18.1 ± 6.3	21.4 ± 8.6
Osteocalcin (μg/L)	5.6 ± 3.4	6.3 ± 3.8

Abbreviations: CTX, carboxy-terminal telopeptide of type I collagen; NTX, urine amino-terminal collagen cross-links; BAP, bone alkaline phosphatase; PTH, parathormone.

**Table 3:** Dual X-ray absorptiometry (DXA) parameters measured in the study groups. Significance was calculated by two-tailed unpaired Student’s *t*-test. For significance: * *p* < 0.05; *** *p* < 0.001.

Parameters	Premenopausal Women (N = 28)	Postmenopausal Women (N = 28)
Lumbar spine BMD (g/cm^2^)	1.232 ± 0.147	1.194 ± 0.173
Hip BMD (g/cm^2^)	1.148 ± 0.146	1.010 ± 0.118 ***
Femoral neck BMD (g/cm^2^)	1.060 ± 0.130	0.917 ± 0.104 ***
Total body BMD (g/cm^2^)	1.272 ± 0.072	1.190 ± 0.109 ***
BMC (g/cm)	2.345 ± 0.434	2.135 ± 0.365
Lean body mass (kg)	53.2 ± 5.1	49.7 ± 6.7 *
Fat body mass (%)	51.7 ± 3.3	53.2 ± 3.1

Abbreviations: BMD, bone mineral density; BMC, bone mineral content.

**Table 4:** Bivariate correlation analysis between sclerostin levels and the main variables of interest in the groups as a whole. Pearson’s correlation coefficient and 2-tailed significance are shown for each variable, with significant associations in bold.

Variables	Sclerostin (pmol/L)	
	r	p
**BMD_LS**	**0.472**	**0.001**
BMD_TH	0.110	0.455
BMD_FN	0.079	0.594
BMD_TB	0.186	0.206
**BMC**	**0.361**	**0.013**
%FBM	0.016	0.915
LBM	0.008	0.955
BMI	−0.017	0.903
Estradiol (μg/L)	−0.090	0.531
**Testosterone (μg/L)**	**−0.290**	**0.041**
25OH-vitamin D (μg/L)	0.088	0.533
PTH (μg/L)	−0.267	0.053
BAP (μg/L)	0.050	0.735
CTX (pg/mL)	0.210	0.161
**NTX (nM/BCE)**	**0.317**	**0.028**
Osteocalcin (μg/L)	0.003	0.981
HOMA-IR	−0.206	0.138
Leptin (μg/L)	0.081	0.565

Abbreviations: BMD, bone mineral density; BMD_LS, BMD at the lumbar spine; BMD_TH, BMD at the total hip; BMD_FN, BMD at the femoral neck; BMD_TB, total body BMD; %FBM, percent fat body mass; LBM, lean body mass; BMI, body mass index; PTH, parathormone; BAP, bone alkaline phosphatase; CTX, carboxy-terminal telopeptide of type I collagen; NTX, urine amino-terminal collagen cross-links; HOMA-IR, homeostasis model assessment-estimated insulin resistance.

**Table 5:** Multivariable regression analysis in merged study groups. Results are provided for variables in the regression equation, adjusted R^2^ values for significant predictors, standardized coefficients (β) and *p* values, with significant associations shown in bold character. For menopause: 0 = no, 1 = yes. For significance: *, *p* < 0.05; **, *p* < 0.01, ***, *p* < 0.001.

Variables	Sclerostin	Lumbar Spine BMD	Femoral Neck BMD	Total Hip BMD	Total Body BMD	Total Body BMC
	*β Value*					
	*(aR^2^, 0.390)*	*(aR^2^, 0.351)*	*(aR^2^, 0.374)*	*(aR^2^, 0.373)*	*(aR^2^, 0.481)*	*(aR^2^, 0.345)*
**Sclerostin**	**-**	0.573 **	0.262	0.209	0.250	0.472 **
**Menopause**	0.395 **	−0.174	−0.561 ***	−0.454 **	−0.344 *	−0.357 *
**Testosterone**	−0.428 **	0.117	0.080	0.012	−0.030	0.040
**PTH**	−0.191	−0.142	0.010	−0.155	−0.055	0.011
**Osteocalcin**	0.149	−0.124	−0.172	−0.185	−0.290	−0.037
**LBM**	0.160	0.085	0.150	0.177	0.353 **	−0.122
**HOMA-IR**	−0.206	0.111	0.002	0.062	0.050	−0.123
**Leptin**	0.061	−0.196	0.075	0.019	0.046	−0.289

For abbreviations: aR2, adjusted R2; BMD, bone mineral density; BMC, bone mineral content; PTH, parathormone; LBM, lean body mass; HOMA-IR, homeostasis model assessment-estimated insulin resistance.

## Data Availability

The datasets generated during and/or analysed during the current study are available from the corresponding author on reasonable request.
